# Beyond the Individual: Couple-Centered Health and Wellness Coaching to Address the Burden of Chronic Conditions

**DOI:** 10.5888/pcd23.260070

**Published:** 2026-08-27

**Authors:** Fariha Niazi, Shazia Akhtarullah

**Affiliations:** 1 Department of Couple and Family Therapy, Dr. Kiran C. Patel College of Osteopathic Medicine, Nova Southeastern University, Fort Lauderdale, Florida; 2 Department of Psychiatry and Behavioral Medicine, Dr. Kiran C. Patel College of Osteopathic Medicine, Nova Southeastern University, Fort Lauderdale, Florida

Approximately 194 million adults live with 1 or more chronic diseases (1). Many of these conditions require ongoing lifestyle modifications in addition to medical treatment and are associated with consequential increases in health care costs and mortality and disability rates (2). Notably, the burden of chronic diseases often extends beyond people to social networks, including partners and families.

Living with chronic disease is shaped by interpersonal processes within family and couple systems (3). When one or both partners are affected, the impact is felt by the couple as a unit. Roles shift, routines change, and emotions are heightened (3). In the presence of chronic disease, health-related changes are negotiated within shared daily routines, roles, and expectations. Lifestyle management, such as diet, exercise, sleep habits, and stress reduction, is not often accomplished alone. Lifestyle management is navigated every day in intimate relationships. Partners frequently engage together in caregiving, household responsibilities, and management of emotional and practical demands (eg, concerns of possible disability, dependence, coordination of treatment).

Emerging evidence suggests that couple dynamics influence health behaviors and their outcomes and that interventions focusing on couples, rather than individuals alone, may be more effective in improving health outcomes (4). Some studies have shown the benefits of couple interventions compared with individual-only interventions (5,6). Although further rigorous research is needed to establish effectiveness, couple-centered approaches show considerable promise (7). Despite the growing recognition of the importance of couple- and family-centered care, a need to further develop these interventions and implement them in chronic disease management remains (8).

This essay highlights the perspective that chronic diseases are experienced within interpersonal contexts and that public health approaches may benefit from greater attention to these contexts. We propose a conceptual framework for couple-centered health and wellness coaching that extends existing coaching competencies by incorporating dyadic processes into behavior change efforts. Involving both partners and positioning health behaviors within shared routines may support consistency, adherence, and long-term sustainability. This framework aligns with public health priorities by addressing behavioral drivers of chronic disease, such as health and lifestyle factors (eg, sleep, stress, diet, exercise).

## Chronic Diseases

Chronic diseases are long-term conditions that may vary both within and between diagnoses in terms of etiology, symptom presentation, severity, trajectory, and patient response. For example, diabetes is often characterized by a relatively stable course requiring ongoing self-management; however, the complexity of care may range from routine lifestyle adjustments to intensive monitoring, medical management of medication, and coordination of services (9). In contrast, heart failure often follows a progressive trajectory with increasing functional decline (10). Chronic pain may range from mild, intermittent discomfort to persistent and debilitating forms and is frequently marked by unpredictability and fluctuating intensity that affects daily functioning and quality of life (11). These variations shape how people and couples manage illness over time. Effective long-term management often requires attention not only to individual behaviors but also to the relational environments in which these behaviors occur.

## Relational Perspectives on Chronic Diseases

Family-based perspectives emphasize that illness affects not only biologic functioning but also communication patterns, shared meaning-making, and relationship dynamics (3,12,13). Within the couple system, chronic diseases can disrupt established routines and roles while also creating opportunities for mutual support and coregulation. Whether these dynamics facilitate or hinder adaptations may depend on how couples interpret illness, distribute responsibilities, and communicate about health-related needs. Many lifestyle behaviors that are the focus of chronic disease prevention programs are embedded within shared environments. Food choices are influenced by shared meal planning and cultural norms; physical activity is shaped by coordinated schedules and energy levels; sleep and stress are often regulated within intimate relationships. Interventions that do not consider these interactional patterns may be less effective at supporting long-term adherence.

## Health and Wellness Coaching Perspective

Health and wellness coaching is a client-centered, evidence-informed process that supports people and groups in achieving health-related goals through behavior change (14). It emphasizes leveraging strengths and resources while aligning with clinical care plans. National board-certified health and wellness coaches function as facilitators rather than clinical providers, supporting goal setting, accountability, and lasting lifestyle change (14).

Coaching has demonstrated potential to enhance engagement, support lifestyle modification, and contribute to chronic disease management (15,16). Many of the areas addressed by coaching — including risk factors, behavior change, and prevention — are already reflected within existing clinical documentation systems, such as the International Classification of Diseases system (16). We propose a couple-centered extension of coaching as a relationally informed, nonclinical approach that engages partners collaboratively. Grounded in established competencies, the framework adopts a strengths-based, whole-person perspective that situates health and wellness within social and interpersonal contexts (17).

## Couple-Centered Health and Wellness Coaching Framework

The proposed framework emphasizes the involvement of both partners in lifestyle change processes. By incorporating dyadic engagement, it seeks to integrate health behaviors into shared routines and leverage interpersonal dynamics to support adherence and sustainability (3–8). Sessions may include joint goal setting, collaborative planning, and structured follow-up, with opportunities for both individual reflection and shared discussion. Coaching interactions are typically delivered in person or via telehealth, in 45- to 60-minute sessions across a series of 10 to 12 meetings.

Consistent with established health and wellness coaching practices, ethical considerations are central and include obtaining informed consent from both partners, maintaining professional boundaries, and ensuring confidentiality in the coaching process (14,17,18). Attention to safety, transparency, and power dynamics is also important (18). When appropriate, referrals to specialized services are made, and collaboration with health care providers is maintained to ensure alignment with clinical care (17). This approach differs from processes in which partners may be included in a supportive role rather than fully engaged in shared goal setting and behavior change. Unlike therapy, which addresses mental health conditions, coaching focuses on optimizing health behaviors and supporting goal attainment (14).

## Mechanisms of Action

This framework is proposed to influence sustained behavior change through several interconnected relational processes. Engaging partners encourages communal coping, in which challenges are approached as shared concerns to promote couple collaboration and support (4). The framework also supports adaptive responses to stress through coregulation within the relationship. Accountability within the partnership can reinforce consistency and follow-through, while shared habits may become integrated into daily routines. In addition, reducing relational stress can create an environment that supports engagement and adherence (19). Together, these processes are likely to position interpersonal dynamics as key contributors to long-term behavior change.

## Public Health and Systems Implications

Health and wellness coaching aligns with public health priorities by addressing modifiable risk factors and supporting sustained behavior change in real-world contexts (20). Evidence indicates positive outcomes across populations and settings, particularly in primary care (15). Extending coaching to include partners may further enhance adherence, improve outcomes, and expand the reach of preventive interventions. Integration into primary care and community settings may be achieved through brief, structured sessions embedded within routine care and follow-up appointments. Interdisciplinary teams can incorporate coaching to support shared goal setting, action planning, and follow-up beyond clinical encounters.

From a policy perspective, incorporating partner-inclusive approaches into preventive and behavioral health services may support reimbursement models, workforce development, and value-based care initiatives. These approaches may also contribute to reduced health care utilization associated with poorly managed chronic disease (19). Telehealth delivery can offer additional opportunities to expand access and scalability by enabling remote sessions, ongoing communication, and shared tracking of health behaviors. These strategies are likely to improve accessibility while maintaining continuity of support for both patients and partners (15).

## Alignment With Chronic Disease Prevention Frameworks

Couple-centered health and wellness coaching aligns with some chronic disease prevention frameworks by embedding behavior change within interpersonal and system-level contexts central to population health. For example, consistent with the social ecological model, it targets the interpersonal level by engaging partners as active participants, recognizing that health behaviors such as diet, exercise, and stress management are shaped within shared environments (21). By incorporating both partners, coaching extends beyond individual-focused strategies to address relational influences that affect adherence and long-term sustainability. Couple-centered health and wellness coaching also aligns with the Chronic Care Model by supporting self-management, patient engagement, and goal-oriented care (22). Through shared goal setting, collaborative action planning, and ongoing follow-up, couple-centered coaching can reinforce key components of effective chronic disease management. From a population health perspective, integrating couple-centered coaching into primary care and community settings may enhance reach, improve adherence, and support prevention efforts that focus on modifiable risk factors (23). By leveraging interpersonal dynamics, this framework offers a scalable strategy to strengthen chronic disease prevention and management while addressing the relational contexts in which health behaviors occur.

Chronic diseases are not experienced or managed in isolation. They are lived within interpersonal contexts that shape daily health behaviors and influence long-term outcomes. Incorporating relationally informed approaches, such as couple-centered health and wellness coaching, into chronic disease management efforts may strengthen public health strategies focusing on improving population health while supporting the lived realities of individuals, couples, and families.
